# Species Specific Differences of CD1d Oligomer Loading *In Vitro*

**DOI:** 10.1371/journal.pone.0143449

**Published:** 2015-11-24

**Authors:** Daniel Paletta, Alina Suzann Fichtner, Lisa Starick, Steven A. Porcelli, Paul B. Savage, Thomas Herrmann

**Affiliations:** 1 Institute for Virology and Immunobiology, University of Würzburg, Würzburg, Germany; 2 Department of Microbiology and Immunology, Albert Einstein College of Medicine, Bronx, NY 10461, United States of America; 3 Department of Chemistry, Brigham Young University, Provo, UT 84602, United States of America; Cardiff University School of Medicine, UNITED KINGDOM

## Abstract

CD1d molecules are MHC class I-like molecules that present glycolipids to iNKT cells. The highly conserved interaction between CD1d:α-Galactosylceramide (αGC) complexes and the iNKT TCR not only defines this population of αβ T cells but can also be used for its direct identification. Therefore, CD1d oligomers are a widely used tool for iNKT cell related investigations. To this end, the lipid chains of the antigen have to be inserted into the hydrophobic pockets of the CD1d binding cleft, often with help of surfactants. In this study, we investigated the influence of different surfactants (Triton X-100, Tween 20, Tyloxapol) on *in vitro* loading of CD1d molecules derived from four different species (human, mouse, rat and cotton rat) with αGC and derivatives carrying modifications of the acyl-chain (DB01-1, PBS44) and a 6-acetamido-6-deoxy-addition at the galactosyl head group (PBS57). We also compared rat CD1d dimers with tetramers and staining of an iNKT TCR transductant was used as readout for loading efficacy. The results underlined the importance of CD1d loading efficacy for proper analysis of iNKT TCR binding and demonstrated the necessity to adjust loading conditions for each oligomer/glycolipid combination. The efficient usage of surfactants as a tool for CD1d loading was revealed to be species-specific and depending on the origin of the CD1d producing cells. Additional variation of surfactant-dependent loading efficacy between tested glycolipids was influenced by the acyl-chain length and the modification of the galactosyl head group with PBS57 showing the least dependence on surfactants and the lowest degree of species-dependent differences.

## Introduction

CD1 molecules belong to a non-polymorphic family of non-classical MHC class I molecules (MHC Ib molecules) [1, 2]. All characterized members of the CD1 family are structurally related to MHC class I molecules and have been subcategorized into group 1 (CD1a-c), 2 (CD1d) and 3 (CD1e) [3, 4]. While all CD1 molecules of group 1 and 2 are known to present lipid antigens to T cells, CD1e rather enhances or modulates antigen-loading of the other CD1 molecules [5]. In contrast to humans and many other species, rodents possess *CD1d* as the only CD1 gene [6, 7]. The α1 and α2 domains of CD1d form an antigen binding cleft substantially different from that of MHC class I molecules: Accessible through a single narrow portal (F’ portal), it is located between both helices where two deep hydrophobic pockets (A’ and F’) are formed beneath the surface of the CD1d molecule [8–10]. This allows insertion of hydrophobic chains and therefore lipid antigen presentation. In this way CD1d of most species is able to present antigens to Type 1 NKT cells (iNKT cells), a “non-conventional” αβ T cell subpopulation [11–15]. This distinct population can be defined by the highly conserved interaction between its semi-invariant iNKT TCR (Vα14/Jα18 paired with Vβ8.2 in mice and Vα24/Jα18 paired with Vβ11 in human) and the cerebroside α-Galactosylceramide (αGC) presented by CD1d [16–20]. A synthetic form of αGC known as KRN7000 is the first characterized and one of the strongest known antigens for iNKT cells. The galactose head group of KRN7000 is α-anomerically linked to ceramide containing a phytosphingosine backbone and a C26:0 acyl chain. During the binding of iNKT TCRs to CD1d molecules presenting αGC, the acyl and sphingosine chains are inserted into the A’ and F’ pockets of CD1d, respectively, while the polar head group protrudes above the antigen binding cleft to make direct contacts with the iNKT TCR [11–14].

This lineage-defining interaction of the iNKT TCR itself allows a specific and direct identification of iNKT cells, and made glycolipid loaded CD1d oligomers an indispensable tool for iNKT-cell related investigations [21–24]. Still, the utility of such reagents strongly depends on effective *in vitro* loading with defined lipid antigens, which is prerequisite of high affinity binding of the semi-invariant iNKT TCR. *In vivo*, CD1d is initially loaded with self-lipids in the ER, facilitated by the microsomal triglyceride transfer protein (MTP), while lipid exchange mostly takes place in acidic compartments of the cell and is realized by different classes of proteins, such as CD1e and saposins [6, 25, 26]. *In vitro* loading of CD1d oligomers is predominantly performed in the presence of surfactants, which facilitate the insertion of the hydrophobic lipid chains into the CD1d binding cleft within an aqueous solution. Previous studies could show how length and structure of the hydrophobic chains influence CD1d loading and therefore the usefulness of CD1d tetramers [27]. The use of different surfactants for *in vitro* loading of CD1d oligomers has been described, but published studies have used CD1d molecules originating from different species whose production followed different protocols [21–23, 27, 28]. In the current study, we have compared loading of CD1d-mouse-IgG1 heavy chain based dimer constructs containing CD1d sequences from human, mouse, rat and cotton rat produced under the same protocol in our laboratory [29, 30] as well as rat CD1d dimers and tetramers. We put focus on the usability of different surfactants in loading of CD1d from different species with structurally diverse αGC derivatives under conditions typically used for *in vitro* loading of CD1d. We found that efficacy of *in vitro* loading was strongly affected by the different surfactants dependent on species-origin of CD1d and type of CD1d producing cell, and modulated by the acyl chain length of the respective glycolipid. In addition, modification of the hydrophilic part of the glycolipid PBS57 allowed its loading without usage of surfactants. Overall, our findings not only demonstrate the pivotal role of CD1d loading efficacy for efficient detection of cell surface expressed iNKT TCR but also the requirement of matching loading conditions and CD1d oligomer/glycolipid combinations.

## Materials and Methods

### Cloning and expression of rat/mouse iNKT TCR

Cloning of mouse *AV14S1A2* TCRα chain as well as rat *BV8S4*-like (CDR2+4) β chain has been described in [31] and [32] respectively. A93G mutation of mouse *AV14S1A2* TCRα chain has been described in [30]. Mouse iNKT TCRα as well as rat β chain were transduced retrovirally and expressed in BW r/mCD28 cells, which are BW58 TCR-negative mouse hybridoma cells transduced with a chimeric rat/mouse CD28 molecule as described in [33].

Cell surface expression of transduced TCR was analyzed by staining with biotinylated anti-mouse CD3ε mAb (BD Pharmingen), revealed by Allophycocyanin conjugated streptavidin (BD Pharmingen). Cells were sorted using a FACS Aria III (BD Biosciences) machine.

### Cell culture

BW r/mCD28 iNKT TCR cells were cultured in RPMI 1640 medium supplemented with 10% FCS, 1 mM sodium pyruvate, 2.05 mM glutamine, 0.1 mM nonessential amino acids, 25 μM β-mercaptoethanol, penicillin (100 U/ml) and streptomycin (100 μg/ml) at 310.15 K with 5% CO_2_ and an H_2_O-saturated atmosphere.

### Lipids and sufactants

α-Galactosylceramide (αGC) was purchased from Avanti Polar Lipids. PBS44 and PBS57 are described in [34, 35]. DB01-1 is described in [27]. All lipids used in this study were dissolved in 100% Dimethylsulfoxide at a concentration of 500 μM by sonication for 5 min at RT. All surfactants used in this study were purchased from Sigma-Aldrich (St. Louis, MO, USA) and diluted 1:100 in PBS.

### Cloning and production of CD1d Dimer constructs

Generation of human, mouse, rat [29, 30] and cotton rat CD1d dimers [36] was performed as described previously for mouse CD1d dimers [37]. Modification such as the use of rat β2-microglobulin transduced J558L cells for rat CD1d dimer production and construction of the CD1d dimer expression vectors have been performed as described in [38]. In short, CD1d extracellular domains α1–3 were amplified by PCR and cloned into the MluI/XhoI sites of the pXIg vector [39], thereby generating a CD1d/mIgG heavy chain chimeric construct with CD1d inserted between mIgG heavy cain leader and variable sequences. CD1d dimer gene constructs were expressed by J558L mouse myeloma cells [40] and purified from culture supernatants as described [38]. CD1d dimers were stored at 4°C in PBS containing 0.02% NaN_3_.

### Rat CD1d tetramers

Unloaded PE conjugated rat CD1d tetramers were generated from CD1d monomers and rat β2-microglobulin produced by human embryonic kidney derived 293T cells (Rick Willis, personal communication) and obtained from the NIH Tetramer Core Facility, Emory University Vaccine Center, 954 Gatewood Road Atlanta, GA 30329, USA.

### Loading of CD1d oligomers

CD1d oligomers (at a final concentration of 250 μg/mL in PBS) were loaded with 40x molar excess of lipid (dissolved at 500 μM in DMSO; final amount of DMSO in loading mixture: 7.5%) by incubation for 22 h at 37°C in the presence of 0.05% surfactant or the corresponding amount of PBS. As negative control, CD1d dimers were incubated with the corresponding amount of DMSO without lipid (vehicle loading).

### Flow cytometry

Cells were analyzed on a FACSCalibur machine (BD Biosciences) using FlowJo software (TREE STAR Data analysis software). CD1d oligomer staining was carried out at room temperature for 30 min in 100 μl of sample containing 10^5^ iNKT TCR transductant cells suspended in FACS buffer (PBS pH 7.4, BSA 0.1%, 0.01% NaN_3_). Bound CD1d dimers were detected with PE labeled donkey F(ab′)_2_ fragment anti-mouse IgG (H+L) with minimal cross-reactivity towards rat and other species serum proteins (Dianova), referred hereafter as DαM. After incubation with DαM, nonspecific binding to the DαM Ab was blocked with IgG from mouse serum and biotinylated anti-mouse CD3ε (clone 145-2C11) was added. Biotinylated antibodies were detected with Allophycocyanin conjugated streptavidin (BD Pharmingen).

### Statistical analysis

CD1d dimer staining was visualized and statistically analyzed using Prism (GraphPad).

## Results

### Production of CD1d dimers and *in vitro* loading analysis

**Fig 1:**
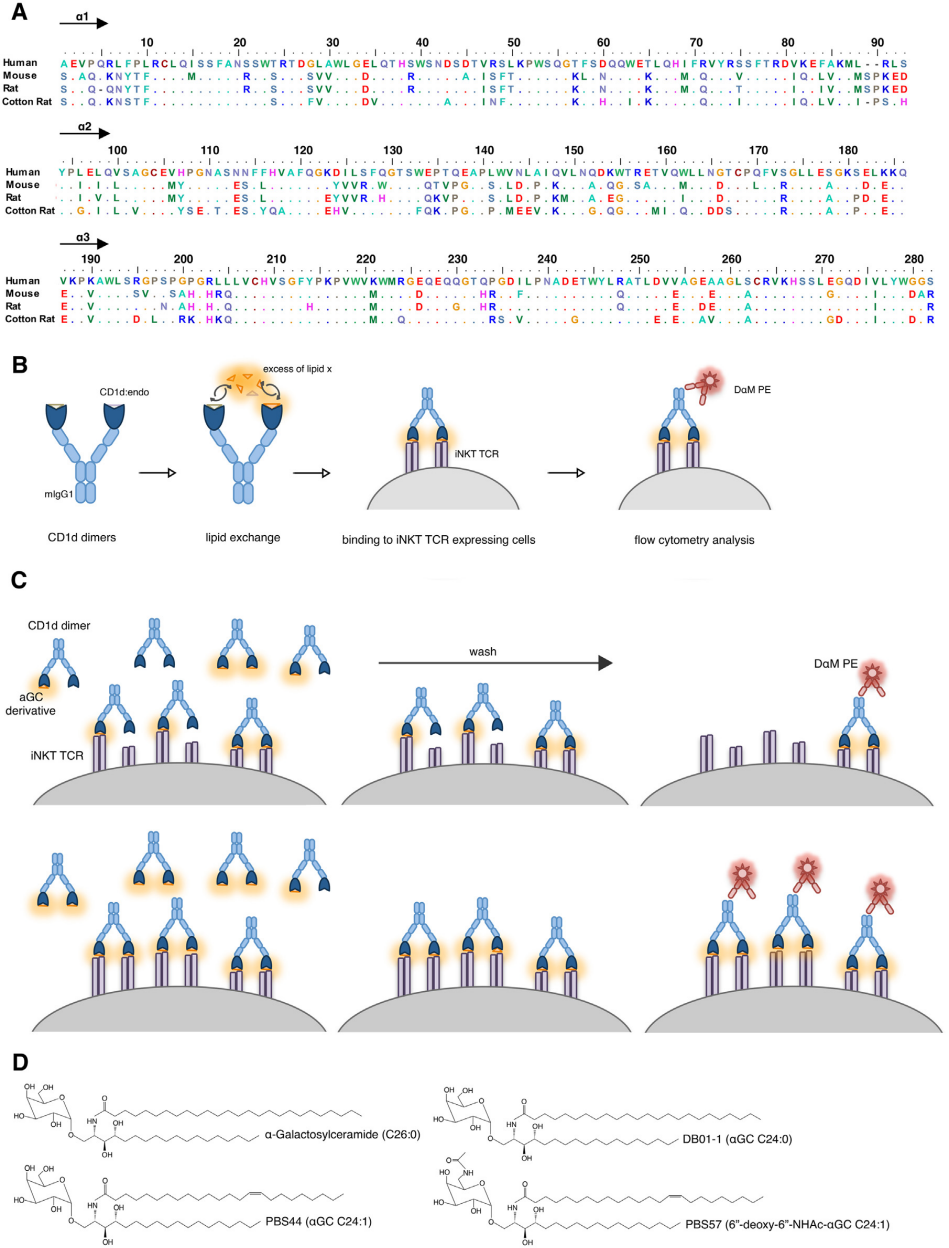
Experimental setup. (A) α1 and α2 domain amino acids of CD1d molecules used in this study. Alignment was performed using BioEdit. Human: GenBank BC027926.1, Mouse: GenBank AK002582.1, Rat: GenBank AB029486.1, Cotton Rat: GenBank KM_267558. (B) *In vitro* loading analysis of CD1d dimers presented as schematic overview. (C) Scheme explaining the emergence of different saturating curves depending on loading efficacy of dimers. Upper panel: Inefficient loading leads to low proportion of functionally bivalent CD1d dimers, Lower panel: Efficient loading leads to high proportion of functional bivalent CD1d dimers. Drawing represents several step flow cytometry analysis of incubation/wash/detection. (D) Structural features of all glycolipids used in this study.

Human, mouse and rat CD1d dimer constructs used in this study have already been described in the literature [29, 30]. Also included in the analysis were CD1d dimers of cotton rat which have recently been generated in our laboratory [36]. Such dimers allowed the characterization of iNKT cells from this species. As found for other muroid rodents cotton rat possesses CD1d as single member of the CD1 family with high sequence homology of its extracellular domain to CD1d of mouse, rat and human (Fig 1A) [36]. The extracellular part of CD1d (Fig 1A; domains α1–3) was cloned 5’ of the V segment of a rearranged mouse IgG1 heavy chain. Cloned CD1d-mIgG1-HC constructs were used for transfection of rat β2-microglobulin transduced J558L mouse B cell lymphoma cells lacking Ig heavy chain expression but capable of producing mouse Ig lambda light chain as well as mouse β2-microglobulin. Supernatant of resulting CD1d-mIgG dimer producing cell lines was collected and dimers were purified. For loading of CD1d, 250 μg/mL dimers in PBS were incubated with 40 times molar excess of lipid antigen o.n. in presence of 0.05% surfactant or the corresponding amount of PBS at 37°C (Fig 1B). Lipid antigens were used at 500 μM in DMSO. Vehicle loading of CD1d dimers as control was performed using corresponding amount of pure DMSO.

We compared the efficacy of CD1d *in vitro* loading with αGC, DB01-1, PBS44 and PBS57 without adding surfactant or in presence of Triton X-100, Tween 20 and Tyloxapol. After *in vitro* loading with respective lipid antigens, CD1d dimers were directly used for staining of transduced mouse T-cell hybridoma cells expressing an iNKT TCR as indicator of effective loading with the respective lipid antigen (Fig 1C). In order to increase comparability of the CD1d binding assays all experiments were performed with the same TCR-transductant. Since CD1d molecules from different species vary considerably with respect to cross-species reactivity (e.g. mouseCD1d binds only poorly to rat iNKT TCR [30]) a transductant with a canonical mouse AV14/AJ18 iNKT TCRα chain paired with a rat BV8S4 iNKT TCRβ chain was used which binds CD1d of tested species with similar efficacy. Binding of CD1d dimers was detected by staining with PE conjugated donkey anti-mIgG F(ab’)_2_ fragment. Staining was performed three times independently using CD1d dimers derived from two independent *in vitro* loadings. Resulting staining is presented as relative to anti-mCD3 staining to compensate for variation in surface TCR expression.

Fig 1C shows structural features of all tested glycolipids. αGC, DB01-1 and PBS44 have an identical head group but differ at the acyl chain: Compared to αGC, DB01-1 is characterized by a shortened acyl-chain (C26:0 -> C24:0) and compared to DB01-1, PBS44 by an additional C-C double bond introduced between C15 and C16 of the acyl chain (C24:0 -> C24:1). PBS57 was the only tested αGC derivative with a modification at the sugar head group: PBS44 and PBS57 share an identical acyl chain (C24:1) but PBS57 bears an additional amide group at the C6 atom of the galactose head group.

All surfactants tested within this study have been described for loading of CD1d oligomers [21–23, 27, 28]. All are non-ionic but differ in critical micelle concentrations (CMC) and hydrophilic lipophilic balances (HLB) (S1 Table).

Therefore, by testing not only successful association of lipids with CD1d but measuring the binding capabilities of CD1d dimers, we could directly analyze their functionality for iNKT cell related investigations and the influence of loading conditions on the usage of CD1d oligomers as a research tool.

### Species-specific differences in surfactant efficacy during loading of CD1d

**Fig 2:**
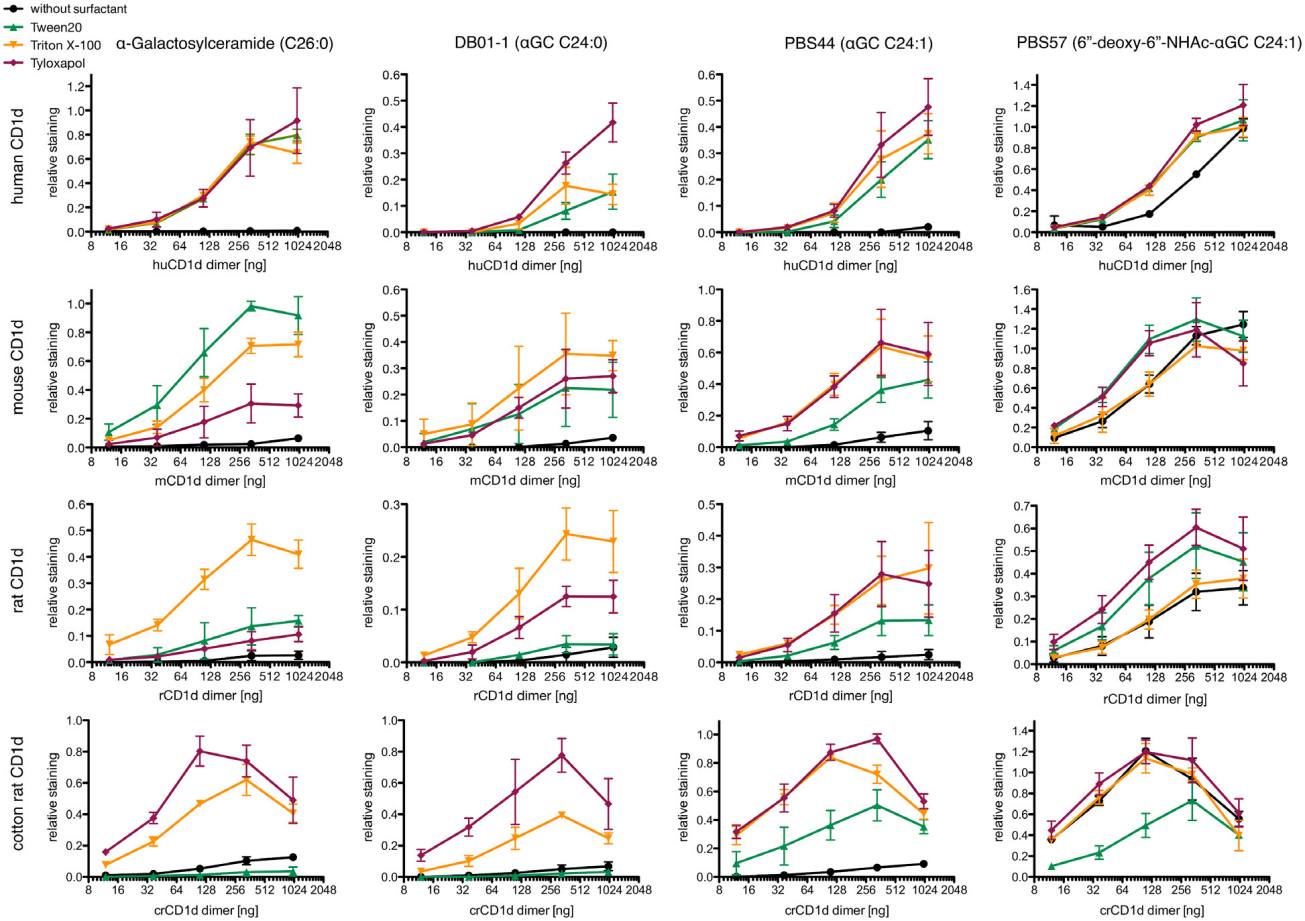
*In vitro* loading analysis of CD1d dimers. CD1d dimers were incubated with 40x molar excess of indicated glycolipid in presence of 0.05% of indicated surfactant or without surfactant o.n. at 37°C. After *in vitro* loading, the indicated amount of CD1d dimers was used for staining of 10^5^ iNKT TCR transduced cells, dimer binding was revealed by staining with PE labeled donkey F(ab′)_2_ fragment anti-mouse IgG (H+L). Binding presented as relative staining, i.e. ratio between geometric means of CD1d dimer and CD3 antibody staining was performed simultaneously. In total, staining was performed three times independently using oligomers derived from two independent loadings. Hierarchy of surfactant efficacy between experiments is shown in S1 Fig. Note that scales differ in order to facilitate visualization different hierarchies of binding caused by the different surfactants.

Depending on the origin of CD1d, the usage of different surfactants led to substantial differences in αGC loading efficacy (Fig 2 and S1 Fig).

For loading with αGC, surfactants were needed to effectively load CD1d of all species. The surfactants behaved similarly with respect to loading of human CD1d but differed in efficacy of loading CD1d of the other species. Triton X-100 was the only surfactant which led to effective αGC-loading of CD1d from all species. Tween 20 was most efficient with respect to loading of mouse CD1d and Tyloxapol for loading of cotton rat CD1d. In this way, all the tested CD1d differed with respect to the surfactants enabling effective αGC loading.

In comparison to αGC the loading of CD1d from human, mouse and rat with DB01-1 was less effective. For mouse and rat CD1d, surfactant preferences were diminished, while opposite effects could be seen for human and cotton rat CD1d.

In comparison to DB01-1, loading of CD1d from all species with PBS44 was more effective and less depending on the choice of surfactant. But the presence of surfactant was still needed for loading of all CD1d species with both DB01-1 and PBS44.

### PBS57 allows loading without surfactant

In general, loading of PBS57 was more effective than loading of any other αGC derivative (Fig 2). Compared to PBS44, loading of PBS57 led to diminished preferences in surfactant usage and changed preferences for rat CD1d where Tyloxapol and Tween20 became more effective than Triton X-100. Interestingly, in contrast to αGC, DB01-1 and PBS44, loading of PBS57 did not require the presence of any surfactant for effective loading of CD1d regardless of species origin. Indeed, for all species the loading efficacy of PBS57 without usage of surfactant was comparable to loading in presence of Triton X-100.

### Glycolipid depending differences in loading efficacy

**Fig 3:**
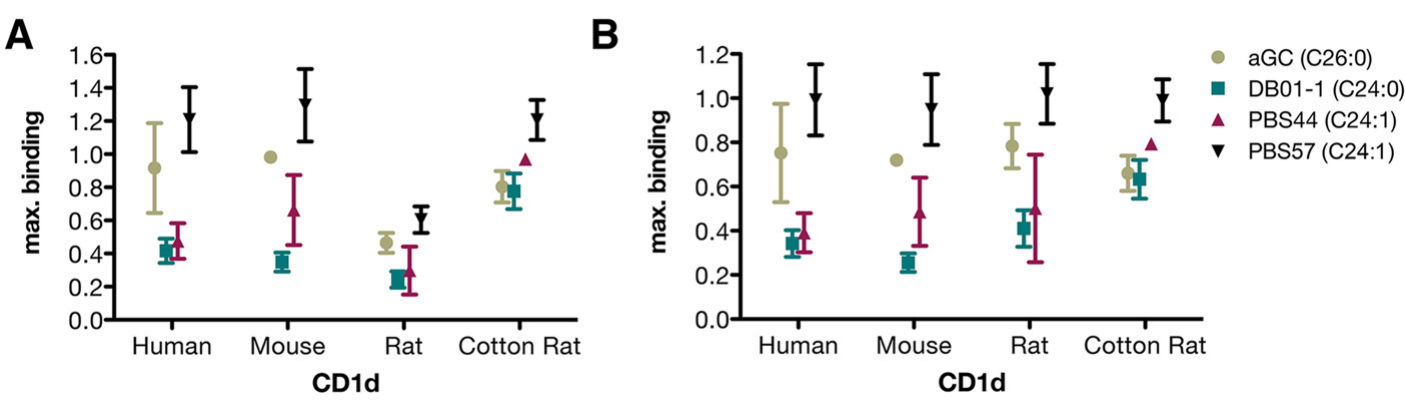
Maximum binding of differently loaded CD1d dimers. (A+B) Highest relative staining reached with each CD1d dimer loaded with indicated glycolipid as presented in Fig 2. Presented (A) as absolute or (B) as relative to the highest value reached for each CD1d. Human CD1d: Binding maxima are for all glycolipids are reached with Tyloxapol as surfactant. Mouse CD1d: Binding maxima with αGC and PBS57 are reached with Tween 20, forDB01-1 with Triton X-100 and for PBS44 with Tyloxapol. Rat CD1d: Binding maxima for αGC, DB01-1 and PBS44 are reached with Triton X-100, and for PBS57 with Tyloxapol. Cotton Rat: All maxima reached using Tyloxapol. Statistical analysis can be found in S2 Table. Hierarchy of surfactant efficacy between experiments are shown in S1 Fig. Data were computed from results shown in Fig 2.

We also compared the maximum binding observed with every combination of CD1d dimer and glycolipid tested in this study independent of the surfactant used for loading (Fig 3A). This analysis showed a similar pattern of glycolipid dependent differences in loading efficacy for human, mouse and rat CD1d: the most effective loading of PBS57 led to the highest binding of CD1d dimers, followed by αGC, PBS44 and DB01-1. We also calculated the maximum binding of αGC, PBS44 and DB01-1 as relative to the maximal binding of PBS57 for each species separately to ignore species-mismatch and TCRα position 93 related CD1d-intrinsic effects [30] on binding of the specific TCR (Fig 3B). In this way the glycolipid depending pattern becomes even more clearly. Statistical analysis showed significance for differences in maximum binding between αGC and DB01-1 as well as between PBS57 and both PBS44 and DB01-1 in human, mouse and rat CD1d (S2 Table). The highest maximum binding of cotton rat CD1d dimers was also observed with PBS57, showing a significantly higher maximum binding than all other tested glycolipids. Loading of cotton rat CD1d with PBS44 was more effective compared to αGC and DB01-1, but only differences between PBS44 and DB01-1 were found to be statistically significant.

### Comparison of rat CD1d dimer and rat CD1d tetramer loading

**Fig 4:**
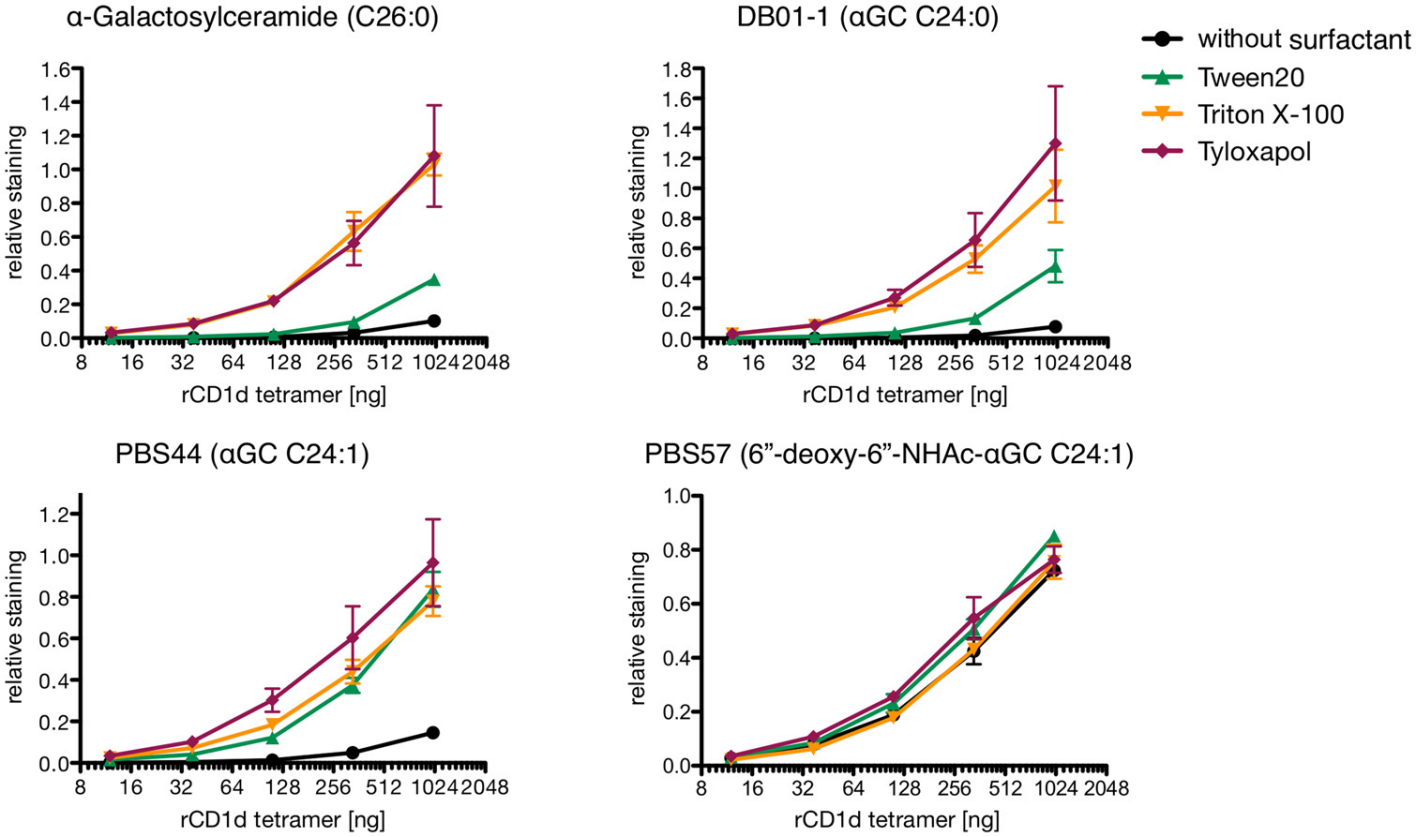
*In vitro* loading analysis of rat CD1d tetramers. PE conjugated CD1d tetramers were incubated with 40x molar excess of indicated glycolipid in presence of 0.05% indicated surfactant or without surfactant o.n. at 37°C. After *in vitro* loading, the indicated amount of CD1d tetramers was used for staining of 10^5^ iNKT TCR transduced cells. Binding presented as relative staining, i.e. ratio between geometric means of CD1d tetramer and CD3 antibody staining performed simultaneously. In total, staining was performed three times independently using oligomers derived from two independent loadings. Hierarchy of surfactant efficacy between experiments is shown in S1 Fig.

Since CD1d tetramers are widely used for studying iNKT cells, we went on to compare loading properties of rat CD1d dimers derived from our own production in mouse myeloma cells and rat CD1d tetramers provided by the NIH tetramer core facility produced from CD1d monomers in human embryonic kidney derived 293T cells and with rat β2-microglobulin (Fig 4). There were two main differences in loading of rCD1d tetramers with αGC (derivatives) compared to rCD1d dimers: a) Preferences in the usage of specific surfactants were diminished. Only Tween 20 showed lower loading efficacy for αGC and DB01-1, while loading of PBS44 and PBS57 remained unaffected from the choice of surfactant. b) In comparison to the myeloma produced CD1d-dimers, Tween20 showed lower efficacy in αGC loading than Tyloxapol which was essentially as effective as Triton X100. c) General loading efficacy of DB01-1 was higher than that of αGC while loading efficacy of PBS57 was lower than that of all other αGC derivatives. Still, PBS57 was the only tested derivative that could be loaded without the presence of any surfactant.

## Discussion

The maximum iNKT TCR binding of αGC, DB01-1, PBS44 or PBS57 loaded CD1d dimers from mouse, rat or cotton rat differed strongly at saturating concentrations. A scheme illustrating the underlying mechanism as proposed by us is depicted in Fig 1C. *In vitro* loading of CD1d dimers with a defined lipid x results in a mixture of three different compounds: CD1d dimers of type a) no CD1d molecule presenting lipid x, type b) one CD1d molecule presenting lipid x and type c) two CD1d molecules presenting lipid x. Hereby, the efficacy of the loading process (depending on the surfactant) alters the ratio between those three compounds. After *in vitro* loading, a defined quantity of CD1d dimers is incubated with the iNKT TCR carrying cells. CD1d dimers of type a (unloaded), which lack the specific antigen will not be able to bind any iNKT TCR. CD1d dimers of type b (one CD1d molecule loaded) will be able to bind single iNKT TCRs, CD1d dimers of type c (both CD1d molecules loaded) will be able to bind two iNKT TCRs at once. The different avidity of type b and c dimers results in different off rates, thereby a higher proportion of type b dimers dissociates and is replaced by dimers of type b or type c during incubation. Washing the cells massively reduces the ratio between of free and cell bound dimers hence the different valency of CD1d:glycolipid complexes will directly result in a variant total amount of CD1d dimers to be detected at the cell surface as consequence of the ratio between type b (high off-rate) and type c (low off-rate). In this way the ratio between type b and type c in solution after *in vitro* loading defines the point of saturation for the binding curve. In principle this problem applies to all types of TCR-staining using oligomers of complexes of antigens- and antigen-presenting molecules but is especially prominent for CD1:glycolipid complexes given the difficulty to assess proper loading of the molecules as it has been show for CD1d in greater detail previously [41].

The relative efficacy of different surfactants in CD1d loading with αGC and derivatives depended on species or cell type origin of the CD1d. During the loading process, the hydrophobic part of the lipid antigen has to be inserted via the F’ portal into the hydrophic pockets (A’ and F’) of the CD1d antigen binding cleft. Since the preferences in surfactant usage were mainly dependent on the origin of CD1d, it can be assumed that differences within the F’ portal or the antigen binding pockets between the different CD1d molecules would be responsible for this finding. Those CD1d dependent characteristics of lipid loading efficacy were found also to be altered using rat CD1d tetramers instead of rat CD1d dimers. While basic structural features of CD1d heavy chain should not differ between both reagents, the fact that they are produced in different cell types is likely to contribute to those differences. The access to the hydrophobic pockets is essential for the loading of lipid antigens. During the folding process of CD1d, an undefined variety of self lipids are loaded into the CD1d binding cleft. Replacement of those endogenous antigens requires removal of the endogenous lipid and insertion of the defined lipid present in excess within the solvent. Differences in glycosylation may well contribute to the properties of the F’ portal influencing loading of self-lipids, the accessibility of the hydrophobic pockets and the requirements for surfactants facilitating the *in vitro* lipid exchange.

Common for all CD1d molecules and also the analyzed rCD1d tetramer was the lack of effective loading with αGC, DB01-1 or PBS44 in the absence of any surfactant. In contrast to this, loading of all tested oligomers with PBS57 was possible without addition of any surfactant. In other words, the general need for the usage of surfactants did depend on the structure of the antigen itself, not on the origin of the CD1d molecule. Not only the accessibility of the CD1d hydrophobic pockets is crucial during the lipid exchange, but also the accessibility of the hydrophobic chains which have to be inserted. Glycolipids such as αGC and its derivatives are amphiphatic molecules, which will form aggregates like micelles within an aqueous solution if present above a critical concentration. In such aggregates, the hydrophobic chains of the molecules will orientate to the inner phase, which is separated from the aqueous solution by the hydrophilic head groups. The formation of such aggregates can be expected to hamper the loading process since the hydrophobic chains of the single lipid are no longer easily accessible. The presence of surfactants can alter the properties of such aggregates thereby improving the accessibility of the hydrophobic chains of single lipids and facilitating the lipid exchange. But the solubility of lipids can also be altered by structural changes influencing the hydrophilic-lipophilic balance. The shortening of the acyl chain (αGC (26:0) to DB01-1 (24:0)) as well as the introduction of a double bond resulting in a conformational change of the acyl chain (DB01-1 (24:0) to PBS44 (24:1)) did not alter the solubility of the glycolipid in a way that allows loading independent of surfactants. In previous studies it was shown that shortening the 26:0 acyl chain of the aGC to a C20:2 acyl chain is needed for effective loading without usage of Triton X-100 [27]. Here we could see the same effect for the addition of an amide group at the C6 of the galactose of αGC without further modification of the lipid chains. Such modifications of the polar head group can lead to an altered HLB and therefore increase the lipid solubility, improving its accessibility as a free monomer, available for loading into the CD1d binding cleft. Another parameter which has also to be considered is the role of the surfactants in facilitating the dissociation of the endogenous glycolipids from the CD1d molecule. Furthermore, since the dynamics in formation of (mixed) micelles, the interaction between different amphiphatic molecules and thus its availability in an aqueous solution are influenced by the respective concentrations, temperature and pH-value, further and more extensive analysis is needed for a better understanding of the physicochemical basis of this process as well as to determine optimal conditions for the loading of any CD1d molecule/glycolipid combination.

The tetramers and dimers used in this study differ in three major points: 1) Tetramers have a higher maximum valency of TCR binding molecules, namely three [42]. 2) Tetramers are directly labeled thus one of the washing step allowing dissociation of insufficiently loaded oligomers is missing (Fig 1C). 3) Both are produced in different cells. The first two points explain the partial leveling of binding. More interestingly is that surfactants differentially affect loading of CD1d dimers and tetramers, especially in case of αGC: Dimers loaded in presence of Tween 20 bind slightly better than those loaded in presence of Tyloxapol while tetramers loaded in presence of Tyloxapol bind about three time better than those loaded in presence of Tween 20. Since in either case the same CD1d heavy chain amino acid sequences were used for production, variations between the CD1d producing cells must account for this effect. As most likely reason we see differences between the endogenous lipids which needed to be replaced by the glycolipids during loading. This can be caused by either different compositions of human vs mouse endogenous glycolipids or by differences in the intracellular lipid loading machinery.

All together, those results show the importance of proper CD1d *in vitro* loading for iNKT TCR binding studies and demonstrate how the efficacy of different surfactants used at typical conditions strongly depends on species-origin as well as production of CD1d, and how it is further influenced by the structural diversity of the glycolipids to be loaded. This clearly illustrates the need to carefully adjust *in vitro* loading conditions for every CD1d/glycolipid combination to obtain comparable results in iNKT binding studies which include more than one CD1d or glycolipid.

## Supporting Information

S1 Fig Hierarchy of surfactant efficacy at binding maximum. Comparison of maximum binding detected for differently loaded CD1d oligomers in three independent experiments shown in Fig 2. L1 = staining from loading reaction 1, L2a/b = staining a or b from loading reaction 2. The column on the very right summarizes the results of the left columns with an identical scale for the Y-axis. Error bars indicate SD.
(DOC)

S1 Table Surfactants used in this study. CMC = critical micelle concentration, HLB = hydrophilic-lipophilic balance
(DOC)

S2 Table Statistical analysis of CD1d maximum binding. (A-D) Analysis of data as presented in Fig 3A. Given Values beneath respective glycolipid indicate mean ± standard deviation of respective staining, numbers in brackets indicate p values. ns: p > 0.05, *p < 0.05, **p < 0.005, ***p < 0.0005, unpaired t-test.
(DOC)

## Abbreviations

αGC: α-Galactosylceramide
iNKT cells: invariant Natural Killer T cells
