# Supplementary material for: Species Specific Differences of CD1d Oligomer Loading In Vitro
Source: PLoS One. 2015 Nov 24;10(11):e0143449. doi: 10.1371/journal.pone.0143449 (PMC4657966; doi:10.1371/journal.pone.0143449)
Supplement: S1 Fig — Comparison of maximum binding detected for differently loaded CD1d oligomers in three independent experiments shown in Fig 2. L1 = staining from loading reaction 1, L2a/b = staining a or b from loading reaction 2. The column on the very right summarizes the results of the left columns with an identical scale for the Y-axis. Error bars indicate SD. (DOC) [file pone.0143449.s001.doc]

**S1 Fig. Hierarchy of surfactant efficacy at binding maximum.** Comparison of maximum binding detected for differently loaded CD1d oligomers in three independent experiments shown in Figure 2. L1 = staining from loading reaction 1, L2a/b = staining a or b from loading reaction 2. The column on the very right summarizes the results of the left columns with an identical scale for the Y-axis. Error bars indicate SD.
