# Supplementary material for: Species Specific Differences of CD1d Oligomer Loading In Vitro
Source: PLoS One. 2015 Nov 24;10(11):e0143449. doi: 10.1371/journal.pone.0143449 (PMC4657966; doi:10.1371/journal.pone.0143449)
Supplement: S1 Table — CMC = critical micelle concentration, HLB = hydrophilic-lipophilic balance (DOC) [file pone.0143449.s002.doc]

**S1 Table.** Surfactants used in this study

|  | Formula | CMC | MW | Used at | HLB | Class |
| --- | --- | --- | --- | --- | --- | --- |
| Triton X-100 | (C_2_H_4_O)_n_C_14_H_22_O | 0.19-0.24 mM | 625 | 0.856 mM | 13.5 | non-ionic |
| Tween 20 | C_58_H_114_O_26_ | 0.059 mM | 1227.54 | 0.448 mM | 16.7 | non-ionic |
| Tyloxapol | (C_15_H_21_O(C_2_H_4_O)_m_)_n_ | 0.018 mM | 298.42 | 1.860 mM | 12-15 | non-ionic |

**S1 Table. Surfactants used in this study.** CMC = critical micelle concentration, HLB = hydrophilic-lipophilic balance
