# Supplementary material for: Species Specific Differences of CD1d Oligomer Loading In Vitro
Source: PLoS One. 2015 Nov 24;10(11):e0143449. doi: 10.1371/journal.pone.0143449 (PMC4657966; doi:10.1371/journal.pone.0143449)
Supplement: S2 Table — (A-D) Analysis of data as presented in Fig 3A. Given Values beneath respective glycolipid indicate mean ± standard deviation of respective staining, numbers in brackets indicate p values. ns: p > 0.05, *p < 0.05, **p < 0.005, ***p < 0.0005, unpaired t-test. (DOC) [file pone.0143449.s003.doc]

**S2 Table.** Statistical analysis of maximum binding fo CD1d dimers

| human CD1d |  | **αGC**  **(C26:0)** | **DB01-1**  **(C24:0)** | **PBS44**  **(C24:1)** | **PBS57**  **(C24:1)** |
| --- | --- | --- | --- | --- | --- |
|  |  | 0.9160  ± 0.2711 | 0.4170  ± 0.07353 | 0.4760  ± 0.1076 | 1.209  ± 0.1962 |
|  |  |  |  |  |  |
| **αGC**  **(C26:0)** |  |  | *  (0.0370) | ns  (0.0592) | ns  (0.2044) |
| **DB01-1**  **(C24:0)** |  | **  (0.0370) |  | ns  (0.4767) | **  (0.0028) |
| **PBS44**  **(C24:1)** |  | ns  (0.0592) | ns  (0.4767) |  | **  (0.0048) |
| **PBS57**  **(C24:1)** |  | ns  (0.2044) | **  (0.0028) | **  (0.0048) |  |

| mouse CD1d |  | **αGC**  **(C26:0)** | **DB01-1**  **(C24:0)** | **PBS44**  **(C24:1)** | **PBS57**  **(C24:1)** |
| --- | --- | --- | --- | --- | --- |
|  |  | 0.9823  ± 0.03402 | 0.3482  ± 0.05726 | 0.6630  ± 0.2111 | 1.295  ± 0.2186 |
|  |  |  |  |  |  |
| **αGC**  **(C26:0)** |  |  | ***  (<0.0001) | ns  (0.0609) | ns  (0.0706) |
| **DB01-1**  **(C24:0)** |  | ***  (<0.0001) |  | ns  (0.0673) | **  (0.0019) |
| **PBS44**  **(C24:1)** |  | ns  (0.0609) | ns  (0.0673) |  | *  (0.0227) |
| **PBS57**  **(C24:1)** |  | ns  (0.0706) | **  (0.0019) | *  (0.0227) |  |

| rat CD1d |  | **αGC**  **(C26:0)** | **DB01-1**  **(C24:0)** | **PBS44**  **(C24:1)** | **PBS57**  **(C24:1)** |
| --- | --- | --- | --- | --- | --- |
|  |  | 0.4646  ± 0.05957 | 0.2433  ± 0.04922 | 0.2975  ± 0.1444 | 0.6049  ± 0.07997 |
|  |  |  |  |  |  |
| **αGC**  **(C26:0)** |  |  | **  (0.01374) | ns  (0.1374) | ns  (0.0715) |
| **DB01-1**  **(C24:0)** |  | **  (0.0077) |  | ns  (0.5718) | **  (0.0026) |
| **PBS44**  **(C24:1)** |  | ns  (0.1374) | ns  (0.5718) |  | *  (0.0321) |
| **PBS57**  **(C24:1)** |  | ns  (0.0715) | **  (0.0026) | *  (0.0321) |  |

| cotton rat CD1d |  | **αGC**  **(C26:0)** | **DB01-1**  **(C24:0)** | **PBS44**  **(C24:1)** | **PBS57**  **(C24:1)** |
| --- | --- | --- | --- | --- | --- |
|  |  | 0.8033  ± 0.09504 | 0.7763  ± 0.1076 | 0.9663  ± 0.04013 | 1.207  ± 0.0696 |
|  |  |  |  |  |  |
| **αGC**  **(C26:0)** |  |  | ns  (0.7610) | ns  (0.521) | *  (0.0104) |
| **DB01-1**  **(C24:0)** |  | ns  (0.7610) |  | *  (0.0457) | **  (0.0099) |
| **PBS44**  **(C24:1)** |  | ns  (0.521) | *  (0.0457) |  | *  (0.0306) |
| **PBS57**  **(C24:1)** |  | *  (0.0104) | **  (0.0099) | *  (0.0306) |  |

**S2 Table. Statistical analysis of CD1d maximum binding.** (A-D) Analysis of data as presented in Figure 3A. Given Values beneath respective glycolipid indicate mean ± standard deviation of respective staining, numbers in brackets indicate p values. ns: p > 0.05 ,*p < 0.05, **p < 0.005, ***p < 0.0005, unpaired t-test.
